# “If you can, change this system” -Pregnant asylum seekers‘ perceptions on social determinants and material circumstances affecting their health whilst living in state-provided accommodation in Germany - a prospective, qualitative case study

**DOI:** 10.1186/s12889-019-6481-2

**Published:** 2019-03-12

**Authors:** Sandra Claudia Gewalt, Sarah Berger, Joachim Szecsenyi, Kayvan Bozorgmehr

**Affiliations:** Department of General Practice & Health Services Research, Heidelberg University Hospital, Im Neuenheimer Feld 130.3, 69120 Heidelberg, Germany

**Keywords:** Refugees, Asylum seekers, Pregnancy, Maternal health, Social determinants of health, Diet, food, and nutrition, Financial support, Public policy

## Abstract

**Background:**

Pregnant women and new mothers seeking asylum are highly vulnerable and have special needs, yet there is dearth of research related to this group in Germany. This paper reports on material circumstances and behavioural factors as social determinants of asylum seekers’ health during pregnancy and early motherhood. The study aim was to gain in-depth insights into these women’s experiences and perceived needs with a focus on material circumstances whilst living in state-provided accommodation in one federal state in Southern Germany.

**Methods:**

A qualitative, prospective approach was taken with individual semi-structured interviews of participants during pregnancy and up to the six-week postnatal assessment, aiming at interviewing each woman twice during pregnancy and once after giving birth. Two female interviewers performed interviews assisted by female professional interpreters on the telephone. Interviews were recorded digitally and transcribed verbatim. An inductive approach was taken to perform content analysis of interview material.

**Results:**

21 interviews were performed with nine women seeking asylum in pregnancy and early motherhood. Content analysis of women’s perceived health-related needs revealed significant health challenges due to considerable constraints in two major themes each with associated categories: a) material circumstances and b) behavioural factors. Participants’ experiences of living conditions included significant challenges in terms of housing and neighbourhood quality e.g. poor hygiene standards with fear of disease and restless sleep due to threats of violence. Consumption potential was severely limited because of a minimal living allowance. Food was a major preoccupation for all participants. Catering services in state-provided accommodation were perceived as unsatisfactory and neglecting religious practices. Institutional food provided adequate calorific intake but participants reported loss of appetite due to bland food, limited variety, little choice and unfamiliar tastes. Self-catering was prohibited further exacerbating this problem.

**Conclusions:**

Pregnant asylum seekers and new mothers living in state-provided accommodation experienced major restrictions related to material circumstances in this study. Key results identified housing and neighbourhood quality, consumption potential and nutrition as social determinants of health which women perceived to adversely affect their health, especially during pregnancy and early motherhood.

**Electronic supplementary material:**

The online version of this article (10.1186/s12889-019-6481-2) contains supplementary material, which is available to authorized users.

## Background

Social determinants of health, defined as “conditions in which people are born, grow, live, work and age” [1], continuously influence both a person’s and a group’s health status at macro, meso and micro levels and are largely responsible for health inequities. After arrival at the receiving country Germany, health and wellbeing of asylum seeking women, namely those who have sought international protection and whose claim for refugee status has not yet been determined [2], are equally subjected to health inequalities based on social determinants of health, especially during pregnancy and early motherhood. The *Conceptual Framework for Action on the Social Determinants of Health* by the World Health Organization (WHO) [3] schematically depicts how social determinants affect people’s health. At the macro level, the national policies have an indirect impact on the health of individuals, for example through social policies in the housing sector. At the meso level, socioeconomic status indirectly affects one’s health via the purchasing power (or lack of) to access health services. At the micro level, material and psychosocial factors affect a person’s health, for example through poor housing conditions and unsecure neighbourhoods. Social determinants of health, intertwining at macro, meso and micro levels, are factors associated with economic and social conditions and may dispose of a strengthening or deteriorating effect on an individual’s health status. Taking such social determinants of health into account when addressing the health and wellbeing of asylum seeking women is of great importance, particularly during pregnancy and early motherhood.

Focusing on the heterogeneous group of asylum seekers, pregnant women and those in postnatal period (up to the first six weeks after childbirth) are highly vulnerable [4–6]. Female asylum seekers are especially vulnerable with regards to pregnancy-related outcomes [5]. Results from a systematic review on maternal health outcomes in migrants, including those who have crossed an international border or left their habitual place of residence within a state [7], showed that compared to the host population, migrant women had a significant disadvantage for the risk of low birth weight, premature birth and perinatal mortality [8]. Furthermore, migrant women were at an increased risk of serious complications during pregnancy [9] and had an augmented risk for poor maternal health outcomes [10]. Studies on asylum seekers’ maternal health revealed an elevated risk of severe maternal morbidity and of maternal and perinatal mortality [11] and conclude that maternal health needs of asylum seekers are complex [5]. A systematic review of systematic reviews found that asylum seeking women have poorer perinatal health outcomes compared to women from other migrant groups, including mental health, perinatal mortality, and premature birth [12]. Asylum seeking women who are pregnant or new mothers are highly vulnerable and at an increased risk for adverse maternal and perinatal health outcomes.

Based on their increased vulnerability, pregnant women and new mothers seeking asylum have special needs. Even though the *Universal Declaration of Human Rights* highlights that everyone has the right to a living standard “adequate for the health and wellbeing of himself and of his family”, comprising housing, food, clothing and medical care [13], poor social determinants of health during the post-migration arrival and integration phase jeopardize asylum seekers’ health. Often, asylum seekers’ living conditions in reception countries are marked by material and psychosocial deprivation [14, 15]. Asylum seekers face very basic and limited socioeconomic conditions whilst living in state-provided direct provision accommodation [15], so-called reception centres [16]. In the course of the asylum application, a transfer to a shared accommodation centre, also mostly state-owned, is organised. In general, accommodation centres are equipped with kitchens and allow for self-catering. Transfers are realised at any time after the official hearing, a personal interview with each asylum seeker about the individual reasons for flight [17]. The interview takes place within two days after the formal application for asylum [18]. Most European Union (EU) member states acknowledge vulnerable persons, including pregnant women and single parents, yet only very few offer separate accommodation, additional intermediate meals, sports or activities outside the centre or cooking facilities [19]. In some EU countries, such as Ireland, it is vividly discussed if this institutional living and institutional food offer an acceptable standard of living, in particular with regard to the right to adequate housing and rights to food and health [15]. As applied in other EU countries, Germany executes policies of direct provision accommodation [20, 21]. This concept of accommodation offers shelter, catering and a minimal monthly allowance in cash, also referred to as “pocket money”, and in kind to cover personal needs [22, 23]. Direct provision accommodation represents an institutional type of living with basic living conditions and catering (inhabitants are prohibited from cooking themselves) [16]. Direct provision accommodation, which is mandatory for asylum seekers [24, 25], offers strongly limited benefits such as basic housing, meals and a minimal monthly allowance in cash to cover personal needs [22, 26]. In Germany, asylum seekers are initially accommodated at a reception centre and subsequently distributed to an accommodation centre. As a consequence of restrictive policies, asylum seekers have no say in the geographic placement during the process of distribution [16]. The duration of the asylum seeking process may take up to six months or longer [18, 27]. In reception centers in Germany, often a high number of people live in confined spaces and certain rules and standards have to be respected [28]. Public policies between federal states, social determinants at the macro level, vary greatly in Germany and lack nationwide attention of the special needs of vulnerable groups [29]. State-provided accommodations cover basic needs of asylum seekers yet special needs of pregnant women and young mothers are not addressed on a national level [29]. Policies addressing the special needs of pregnant women and new mothers seeking asylum and living in state-provided reception centres vary considerably both within Europe and Germany.

Furthermore, housing and neighbourhood quality are relevant social determinants of asylum seekers’ health as they usually live in state-provided housing within the first weeks and months after their arrival in receiving countries. Poor material circumstances are characterized by a low housing and neighbourhood quality, including a lack of privacy and safety of the living environment [30]. A recent mixed-methods systematic review found consistent correlations between the physical aspects of living conditions and the physical and mental health of asylum seekers [31]. A safe living environment and neighbourhood is especially relevant for single women travelling alone or with children and pregnant and lactating women as they are in need of organised and operational safeguard measures [32]. In receiving countries, many asylum seekers experienced depriving housing conditions and insecurity [33, 34]. Due to the policy of sharing the accommodation with unknown persons, privacy is almost lost [16]. Poor housing was identified as negatively affecting asylum seekers’ health and wellbeing through a range of pathways while improving quality and security of the housing could ameliorate their health outcomes [35]. A study on female refugees in Germany showed that women considered institutional living with overcrowded space and limited privacy without any opportunities for retreat to be burdensome [36]. In Germany, only some federal states accommodate vulnerable groups including pregnant women in separate facilities [29]. A study on asylum seekers in the Netherlands found that poor housing conditions, including privacy, housing and safety had a high influence on their physical health and quality of life [37]. Poor housing and neighbourhood quality, representing important social determinants of health at the micro level, can adversely affect the health of asylum seekers, especially of pregnant women and new mothers.

Furthermore, limited consumption potential displays an added social determinant of health. A large study conducted with humanitarian migrants in Australia found that almost two-thirds suffered from economic problems [38]. They considered financial constraints as a key post-migration stress factor with negative consequences for their health [38]. In Sweden, stressors linked with social and economic burdens have been attributed to adverse health outcomes in asylum seekers [39]. The stress-related effects of socioeconomic worries on the health of refugees are significant [40]. The United Kingdom National Health Service has attempted to tackle this problem by providing additional assistance to pregnant women or those with children under the age of three [41]. Small monthly financial allowances and limited influences on socioeconomic conditions and autonomy, such as the prohibition of self-catering, jeopardize asylum seekers’ health during pregnancy and early motherhood.

In Germany, there is limited evidence of health and health-related experiences and needs of the particular group of asylum seekers who are pregnant or new mothers and live in state-provided accommodation [42]. Therefore, we aimed at gaining in-depth in-sights into asylum seeking women’s experiences and perceived needs during pregnancy and early motherhood, paying special attention to material circumstances in one federal state in Southern Germany.

## Methods

### Design

With the aim of gaining an in-depth understanding of the experiences and perceived needs of asylum seeking women during pregnancy and early motherhood we applied a qualitative methods approach and chose an explorative case study design [43] as the research methodology. A widely used definition of case study research was first described by Yin in his original text *Case study research: design and methods. 1st edition* from 1984 which was revised in 2014 [43]:

A case study is an empirical study that

- investigates a contemporary phenomenon (the “case”) in depth and within its real-world context, especially when
- the boundaries between phenomenon and context may not be clearly evident (p.16).

Another reason why this methodology was considered appropriate was the associated fieldwork as data collection in case study research allows researchers to “get close to the case being studied” ([44], p. 24). In this study, the “case” was defined as: *the experiences and perceived needs of asylum seeking women during pregnancy and early motherhood* examined in the specific context of state-provided accommodations for asylum seekers in Southern Germany.

This study is a prospective study, as we have interviewed women twice during pregnancy and once after giving birth. Being part of a larger study on pregnant asylum seekers and new mothers living in state-provided accommodation in one federal state in Germany, research results on psychosocial factors including future uncertainties, stressful living circumstances and stressful relationships as social determinants of health affecting women’s health and wellbeing have been published elsewhere [45].

### Setting

We conducted this study in one federal state in Southern Germany. According to administrative dispersal quota, this state has received more than 10 % of the total initial asylum applications in Germany in 2016 [46]. During the process of the application for asylum in Germany, asylum seekers have to reside at a state reception center [47]. After having had their official hearing, an interview which is relevant for the further course of the asylum seeking process, state authorities organise transfers to accommodation centres. In this study, participants were living in two out of ten state-owned reception centers. All state-provided accommodations were located in one federal state in Southern Germany, which extends across an area of 35.000 km^2^ and a total population of over ten million inhabitants (comparable to the population size of Belgium or Switzerland).

Reception Center A included ten residential units accommodating over one thousand persons. Study participants lived in five of the ten housing units (Reception Center A-1 to Reception Center A-5). One residential unit (Reception Center A-4 which was later replaced by Reception Center A-5) was reserved for vulnerable groups including pregnant women from the late prenatal period (36th week of pregnancy) or earlier if considered a high-risk pregnancy. If no transfer occurred in between, accommodation in a state-provided reception centre was provided until the end of the postnatal period. Vulnerable groups also comprised male and female disabled persons. Reception Center B also accommodated more than one thousand people. It offered a separate accommodation for single women with or without children. Both reception centers offered rooms with a total of six bunk beds in each room. Reception Center-A and Reception Center-B offered weekly midwifery consultations during pregnancy and early motherhood.

### Participant recruitment

Prior to the onset of the study, the research team established a working relationship with the head of each midwifery team at Reception Center A and B. During a “kick-off meeting” both research team and the midwives talked about the planned research and addressed open questions. For the recruitment process, members of the research team accompanied midwives during consultations in the period from March to May 2016 and from May to July 2017 once a week at both reception centres. During midwifery consultations, these research team members were given the opportunity to talk to asylum seeking women and to invite them to participate in the study. In conversations with potential participants, the research team members described their professional background and informed the women about the study. An information sheet in Arabic, English, Farsi, French, German, Kurdish and Serbian was handed out to potential study participants. Potential participants were informed about the aim of the study, data protection, data usage and potential benefits or risks associated with study participation. In total, 32 women received invitations and nine women provided their informed consent for study participation.

### Sampling strategy

A purposive sampling strategy [48] with intentional selection of female study participants was employed. Striving for “maximum variation” [49], namely a high level of diversity in terms of language and nationality, we sought to include study participants from different geographic origins. We included only pregnant asylum seekers in the first or second trimester of pregnancy to have time for a second interview during pregnancy after at least four weeks between the first and second interview whilst investigating women’s experiences during the asylum procedure over time.

### Data collection

#### Field access

For this study, access to state-provided direct provision accommodation was required. The research team sought and gained field access through official channels via local authorities such as regional councils and local health authorities.

#### Researcher characteristics

The core team consisted of two researchers: SCG and KB, supported by a student, Esther Rottenburg. KB is a male medical doctor with additional qualifications in public health and social epidemiology. SCG is a female medical doctor with additional qualifications in international health and has worked and lived in Southeast Asia and Sub-Saharan Africa. Ms. Rottenburg is a Masters-level Global Health graduate who has worked and lived in Southeast Asia. SCG and Ms. Rottenburg had the opportunity to sharpen their awareness related to cultural differences whilst living in Southeast Asian and Sub-Saharan African cultures. SB, a female with a migrant background herself, joined the research team at the data analysis stage and contributed her expert knowledge in qualitative research. JS is a senior medical researcher with intercultural competencies. All five researchers had carried out studies using qualitative methodology in the past.

#### Interviews

We collected data by performing semi-structured individual interviews. Two female interviewers, SCG and Ms. Rottenburg, started and ended each interview with open-ended questions that allowed participants to address topics that were of importance to them. Interviews were conducted using a semi-structured interview guide and were recorded digitally. In addition, the interview guide for the three follow-up interviews included the following topics: General wellbeing and personal situation; physical and mental health; social wellbeing; pregnancy; medical care in pregnancy; living environment; behavior/ independency; personal characteristics; additional themes raised by the interviewee. During the second and third interviews, these topics were expanded to include the question: What happened since the last interview? Further topics during the third interview were: Delivery and inpatient treatment. Qualitative interviews were performed without an underlying framework. All three detailed interview guides are attached as Additional files 1, 2 and 3. The continuity of the interviewers and interpreters during the follow-up interviews facilitated the establishment of trust, acceptance and a sense of familiarity. Eight women accepted the digital voice recordings of the interviews. One study participant refused digital voice recordings. In this case, field notes and an interview summary were written after each interview.

We arranged place and time for the interview according to participants’ preferences. The interview schedule is shown in Table 1. Interviews were carried out in state-provided direct provision accommodations, also called reception centres, in rooms that allowed for privacy and that were selected in accordance with interviewees. Before the beginning of each interview, participants were asked if they felt comfortable in the venue. We sought to conduct three interviews with each study participant: two interviews during the course of pregnancy and one in early motherhood. A first interview was conducted with nine asylum seeking women during pregnancy. At the time of the first interview, seven out of nine women lived in Reception Center-A, whereas two women lived in Reception Center B. Interviews were performed both at the Reception Centers (Reception Center A-1 to Reception Center A-5 and Reception Center B) and in three different accommodation centers. In this study, women were not generally housed separately from men. At Reception Center A, one housing unit was reserved for vulnerable groups. This housing unit, Reception Center A-4, during the course of the study replaced by Reception Center A-5, sheltered pregnant women with a high-risk pregnancy and any pregnant women in their late prenatal stage (36th week of pregnancy). Women stayed there until the end of their postnatal period, if no transfer took place in between. Before the 36th week of pregnancy, pregnant women were placed in standard accommodations shared with men. As the vulnerable groups also included the disabled, accommodations were not only occupied by women, but also by male asylum seekers. Reception Center B offered a separate accommodation for single women with or without children. Men were not allowed to access this accommodation. Families, including father, mother and one child or several children, have been accommodated in a standard accommodation.

**Table 1:** Study participation and accommodation

	SP1	SP2	SP3	SP4	SP5	SP6	SP7	SP8	SP9
T 1*	x	x	x	x	x	x	x	x	x
Housing Unit	RC_A_1	RC_A_1	RC_A_2	RC_A_3	RC_A_3	RC_B	RC_B	RC_A_4	RC_A_5
T 2*	x	x	x	–	x	–	–	–	–
Housing Unit	AC	AC	AC	–	RC_A_3	–	–	–	–
T 3*	x	x	x	x	x	x	x	–	x
Housing Unit	AC	AC	AC	RC_A_3	RC_A_3	RC_B	RC_B	–	RC_A_5

AC - accommodation center; RC - reception center; SP - study participant; T 1 * - Interview 1 (prenatal); T 2 * - Interview 2 (prenatal); T 3 * - Interview 3 (postnatal)

At the end of the first interview, we asked participants if they allowed us to contact and invite them for a second and third interview using a mobile phone number. All nine women indicated their willingness to participate in future interviews planned in this study. We performed a second and final interview with five women after delivery due to the advanced stage of pregnancy. We lost one study participant from the study as she was not available after the first interview. We interviewed a total of eight women after delivery. We considered data saturation to be achieved when concurrent data analysis did not reveal any new themes arising during the sequence of follow-up interviews.

#### Interview languages

According to the participants’ preferred language, interviews occurred in Albanian, English, Kurmanji and Macedonian. For all interviews that were not conducted in English, female professional interpreters assisted the female interviewers via telephone. All follow-up interviews were realised with the same female interpreter in order to ensure the continuity of the interview setting during the data collection period.

As the research team was mainly German speaking as a primary language, interviews conducted with a female interpreter were performed in German as source language. Therefore, these interviews were transcribed in German and only translated into English where required (usually for publication). Field notes were made in German.

### Data analysis

Transcriptions formed the basis for qualitative content analysis. We used the transcription software f4 version 5 [50] for verbatim transcriptions of digital recordings of interviews. Qualitative data analysis was supported by the software MAXQDA version 12 [51]. We realized data analysis inductively, according to themes that women considered of greatest relevance to them. At the stage of data analysis, we aimed at embedding the findings in an existing theoretical framework. Therefore, themes that emerged during interviews were matched with a suitable framework for analysis. Due to the match between reported experiences of women and the WHO framework on Social Determinants of Health, we considered that framework suitable for our analysis. Data analysis was performed with an inductive approach to thematic analysis. According to Braun and Clarke, we read and re-read the transcripts for both emergent themes (inductive approach) and subsequently for themes related to the WHO framework (deductive approach). Through this intensive analysis process, we generated initial codes, reviewed the transcripts again, reviewed and refined codes, generated shared definitions for codes and grouped the codes via their relationships into categories and overarching themes [52]. This resulted in a finalized consensus-based report. Inductive coding occurred in an iterative process and involved three steps: 1) identifying patterns by open coding of the data, 2) defining thematic categories and 3) establishing core themes. In the first step, there was open coding of appearing and repeating themes. In the next step, we structured these themes by identifying associations with one another and in the last step, we performed selective coding to organise the data into core categories [53]. At each step, the team conducted consensus discussions related to content analysis. To incorporate the results of the ongoing data analysis into the following data collection, both data collection and analysis were carried out in a spiral manner [54]. Therefore, the interview guide was modified and adapted throughout the study. Inductive data analysis based on women’s narratives produced codes and themes parallel to categories in the WHO *Conceptual Framework for Social Determinants of Health* [3]. As a consequence, this framework was drawn upon as a useful conceptual model for the presentation of results to a broad readership.

### Techniques to foster trustworthiness

We complied with the following criteria to maximize trustworthiness of the data: (a) communicative validation, (b) triangulation, (c) validation of the interview situation, and (d) authenticity and trustworthiness; according to Flick et al. [55]. We strived for *communicative validation* (a) by paraphrasing, summarising and validating the given information in the course of the interviews. Applying *investigator triangulation* (b), we aimed at reducing “selective perceptions and blind interpretive bias” [44]. Two persons performed data analysis and coding separately. Additionally, a third party supported consensus discussions. We strove for *source triangulation* (b) by purposive, namely maximum variation sampling of interviewees’ geographic origins [56]. Continuity in the composition of the female team members and female interpreters during interviews was aiming at *validating the interview situation* (c). The interviewers sought to create an atmosphere of trust, understanding and openness to facilitate the interviews by responding to participants’ needs in a sensitive way. Interviewers demonstrated attention and respect for the culturally divergent practices and attitudes of participants during pregnancy and early motherhood to promote *authenticity and trustworthiness* (d). In addition, debriefing discussions and team discussions on findings enabled an in-depth reflection on content and situations and promoted the researchers’ self-awareness during the study. Field notes from each interview facilitated to create a systematic audit trail [57]. Furthermore, the consolidated qualitative reporting criteria (COREQ) [58] and the standards for reporting qualitative research (SRQR) [54] were applied to foster trustworthiness.

## Results

We conducted a total of 21 semi-structured open-ended interviews with nine female asylum seekers during pregnancy and early motherhood between March 2016 and July 2017.

### Sample characteristics

We interviewed a total of nine participants using follow-up interviews during pregnancy and early motherhood. Applying a purposive sampling strategy, we used maximum variation sampling in respect to the geographic origin of study participants. We therefore purposively sampled four asylum seekers from West Africa, three from East Europe, one from West Asia and one from South Asia. At the time of the first interview, participants lived one to six months in Germany and were transferred up to four times between state-provided reception centres at the third and last interview. The participants were between 22 and 37 years old. We provided the study participants’ age in age ranges as the combination of identifiers such as age, geographic origin, pregnancies and marital status might compromise participants’ anonymity (see Table 2).

**Table 2:** Participants’ profiles

	SP1	SP2	SP3	SP4	SP5	SP6	SP7	SP8	SP9
Age range (y)	30–34	35–39	20–24	30–34	35–39	25–29	20–24	30–34	35–39
Time (m)	2	1	1	3	5	6	3	5	2
Transfer (n)	2	4	2	2	1	2	2	1	1
School education	High school	High school	Grade school	none	High school	High school	Grade school	Secondary high school	High school
Geographic origin	West Africa	West Africa	West Asia	East Europe	East Europe	East Europe	West Africa	South Asia	West Africa
Pregnancies (n)	1	1	2	1	2	1	1	2	2
Children (n)	0	0	1	0	1	0	0	1	1
Marital status	S	S	M	M	S	M	S	M	S

*M – Married; S – Single; SP – Study Participant; Time - Months living in Germany at 1st interview; Transfers - Number of transfers until 3rd interview (n)*

Participants had come to Germany one to six months before the first interview. They were accommodated in different state-provided accommodations and transferred up to four times as it is displayed in Table 1. For five out of nine women it was their first pregnancy (see Table 3).

**Table 3:** Themes and categories: material circumstances and behavioural factors

Themes		Categories
Material circumstances	Housing Quality	• Sleep • Hygiene
	Neigbourhood Quality	• Insecurity
	Consumption potential	• Living allowance • Lack of autonomy
Behavioural factors	Nutrition	• Catering
	Physical activity	• Participation

### Themes and categories

The aim of the study was to gain in-depth insights into the experiences and perceived needs of asylum seeking women’s experiences and perceived needs during pregnancy and early motherhood, with a particular focus on material circumstances and behaviors. Based on interviews with study participants, seven categories emerged out of our inductive analysis. It was subsequently identified that these categories could be directly associated with specific themes in the World Health Organization’s *Conceptual Framework for Action on the Social Determinants of Health* [3] i.e.: a) housing quality, b) neighbourhood quality, c) consumption potential, d) nutrition and e) physical activity. These have been grouped into relevant themes from the WHO framework. Inductive analysis and categories are displayed in Table 3.

### Material circumstances: Housing quality

Interviewed women considered living conditions as onerous and unsteady. They reported poor sleep due to high background noise, especially at night. This was perceived as negatively affecting their health and wellbeing:

“Sleeping is not very good, so at night it is definitely difficult… because of the accommodation in general… I don’t feel at ease and the whole back and forth [transfers], I don’t fall asleep quietly.” (SP3_I1).

Whilst being accommodated in state-provided reception centres, difficulties that were inherent in the system were perceived as difficult to bear, especially for pregnant women. In particular, the poor hygiene conditions of the toilet facilities were consistently reported. As a consequence of the high level of contamination, women worried about the healthy course of their pregnancy with toilets being a possible source of infection and felt that the problem of pregnancy-related nausea exacerbated. Shared sanitary facilities in the corridor exacerbated the difficulties experienced by women whilst living in state-provided accommodation. This caused one woman to ask for a change of the system, the camp system, how asylum seekers refer to reception centres in general:

“I can’t believe it. So many dirty toilets. Sometimes vomit… Yeah, very very difficult. I can’t understand how to explain you… If you can, change this system, that camp system. You try it. I think, food, accommodation... because we are women, we can catch infection. Especially pregnant. Very difficult.” (SP8_I1).

Pregnant women and new mothers seeking asylum experienced poor housing quality within the direct provision accommodation. They perceived negative effects for their health and described unmet basic needs including food, sleep and hygienic conditions, which hindered them from feeling at ease whilst living at state-provided facilities.

### Material circumstances: Neighbourhood quality

In addition to the unpleasant housing situation, women described the challenging situation of feeling insecure and having to deal with a lack of personal privacy in the state-provided reception centres. They explained that they could not establish any private sphere for themselves and considered the accommodation as difficult because their room was not lockable and they had to share it with unknown and at times aggressive persons:

“…because I couldn’t sleep that night…I saw that she [roommate] became more aggressive…and she was shouting and throwing things…So when the security came she was reacting, they even called the police, she almost broke teeth… they [security] acknowledged the problem and they [authorities] are going to transfer me.” (SP2_I1).

Being housed at the direct provision accommodation, with no influence over room occupancy or levels of privacy, women stated to feel very anxious, particularly at night. Sharing the room with an unknown woman, several women or even a family, depending on the allocation of other asylum seekers, added to women’s perceptions of limited privacy and security. Those four women in our study who applied for asylum with their husband (and in two cases with their child) were accommodated together with their family members in one room. They reported that the only way of securing themselves was to call the security staff. Reported experiences with the support and protection by security staff were mainly positive.

### Material circumstances: Consumption potential

Study participants reported financial constraints with perceived negative consequences for their health. These were based on restricted monthly living allowances that were largely considered as very little or insufficient and which in case of a change of accommodation were not disbursed. This resulted in a perceived limited consumption potential:

“They give a pocket money and we can buy food from supermarket. But they give small pocket money. One person for 101 Euro. I think, it’s not enough for month …And it’s very difficult. One month we’re waiting for pocket money…After I changed my [location] they didn’t give pocket money. Last month very difficult. Because I have no money.” (SP8_I1).

Participants explained that they are not allowed to cook whilst living at the reception centre. They strongly criticized the experienced lack of autonomy, which was restricting their very basic activities of daily living such as self-catering:

“I would like to heat my food whenever I feel like eating, not... you know? Sometime some people are hungry different time. I just would like... there is a microwave there I just go and heat it and eat… ”(SP2_I1).

In addition to perceiving regulations at state-provided accommodations as restrictive, participants felt they had little or no power to influence or change these conditions which caused women to feel frustrated and desperate about their current situation:

“To be honest I am not satisfied with food, we always receive the same, some soups that I cannot describe, but we have to tolerate it.” (SP6_I1).

Pregnant women and new mothers shared the experienced difficulties they face on a daily basis due to financial difficulties and the restrictive policies which they as asylum seekers have to bear. Participants considered the ability to influence their living situation as minimal.

### Behavioural factors

Pregnant women and new mothers interviewed in this study consistently expressed their disappointment with the accommodation’s catering. Vivid descriptions of the tasteless food, perceived poor quality and lack of variety were given. A big problem was that catering was felt to be insufficient whilst being pregnant or a new mother. One woman pointed out that the only way of enduring the situation at the reception centre is to accept it in order to survive:

“…you just have to survive. For dinner is nothing. It’s only breakfast and lunch. There are only two times [meals], breakfast and lunch. When they give you, it is one [portion], is one for the day. One portion of breakfast for the day, one portion of lunch for the day. That’s why I buy these cornflakes. They give us milk every morning. [In the evening] eat cornflakes or bread. It’s not enough but I have to cope.” (SP1_I1).

Furthermore, women expressed their dilemma of wishing to adhere to their specific cultural and religious practices with respect to the consumption of food and drinks but lacking the sufficient knowledge of the catering services’ standards:

“There is enough food but the food is not tasty. There is fruit yes, vegetables almost none, mainly fruit… We don’t know if the food is halal but we eat it.” (SP3_I1).

Another topic of importance to interviewed women was related to physical activities. While waiting for their asylum seeking request to be processed, women reported the lack of opportunities and information on exercise and movement that would be beneficial for them as a pregnant women:

“Yeah, I if possible I would like to would have the information. I know there are some exercises on places you go for exercise as a pregnant woman. If I can have that I would be happy.” (SP5_I2).

Study participants shared their perceptions of unmet needs for a healthy and nutritious diet and sound physical activities while being housed in the reception centres. This was considered to be a great concern for their personal health and wellbeing and for that of their unborn child.

## Discussion

This exploratory case study offers in-depth insights into the experiences and perceived needs of the vulnerable group of pregnant women and new mothers seeking asylum whilst living in state-provided accommodation in one federal state in Southern Germany. Focusing specifically on material circumstances and behavioural factors as social determinants of health, the main findings of this study showed that these factors exerted a perceived negative impact on the health and wellbeing of the women.

Study participants were transferred up to four times between state-provided reception centers and expressed a high level of dissatisfaction with their overall living conditions whilst being accommodated at reception centres. Their frustration was linked to the housing and neighbourhood quality which, according to interviewees, lacked privacy and security. They reported that sharing a room with unknown and sometimes intrusive persons negatively impacted on their feeling of security and privacy and resulted in poor sleep quality. Women also stated that due to prevailing regulations they had no influence over the occupancy of their room. Additionally, pregnant women described the exacerbation of their pregnancy-related nausea and their fear of catching an infection due to the poor hygienic conditions at their accommodation. The pregnant women and new mothers perceived a poor standard of housing and neighbourhood quality, a social determinant of health at the micro level, whilst being accommodated at state-provided reception centres as detrimental to their health.

Another aspect that added to the asylum seeking women’s discontent with the state-provided accommodation, which they considered as highly challenging, was the perceived dearth of consumption potential as a result of the restricted financial living allowance. They reported hardship when left without any money if their monthly allowance in cash was late or omitted, e.g. in case of a relocation. Moreover, limited financial resources meant the women could not afford to buy much of their own food and were reliant on the basic catering services. The prohibition to cook in reception centres was considered as adversely affecting their nutrition and wellbeing. According to them, the catering’s bland food with little choice was considered to be insufficient in terms of quality, especially concerning the particular needs during pregnancy and early motherhood. Asylum seeking women in our study also criticized that catering neglected individual dietary needs and cultural and religious practices. In addition, the women had limited information and availability of physical exercises recommended for pregnant women at the direct provision accommodation. Most interviewed women expressed their frustration related to these conditions stating that they had no other possibility than to accept and endure the conditions in the so called “camps” in order to “survive”. Only one participant asked for a change of the system. In our study, pregnant women and new mothers experienced restrictive regulations that negatively impacted on their material circumstances and perceived health in state accommodation.

The discussion about changing the system of state-provided reception centres with restrictive and even repressive regulations limiting asylum seekers’ autonomy is subject to a lively debate in reports and studies. A study on asylum seekers in an eastern state in Germany emphasized on the perceived negative consequences of repressive regulations and various socio-environmental challenges during the asylum seeking process [59]. Based on a study of accommodation centres in the Czech Republic, a report by the International Organization for Migration labelled these centres as long-term “confinement” and “tools of migration control”, stating that in reception centers, control and assistance go hand in hand, creating a repressive environment for asylum seekers [60]. In the United Kingdom, which also applies policies of direct provision accommodation, asylum seekers are often accommodated in areas that are coined by deprivation, with the consequence of social determinants of ill-health [61]. The necessity of remaining at a particular allocated accommodation during the asylum application exacerbates the adverse consequences [61]. Research on reception conditions for asylum seekers in the Republic of Cyprus labeled the circumstances as inadequate and problematic [62]. Addressing the restrictive regulations at state-provided reception centres, a social determinant of health at the macro level, might be beneficial for asylum seekers’ health, especially during periods of increased need including pregnancy and early motherhood.

The lack of autonomy in state-provided direct provision accommodation is further exacerbated by asylum seekers’ experiences of a limited consumption potential. Women in our study highlighted the harmful consequences of financial deprivation for their health and wellbeing. Poverty and insufficient financial means impact adversely on an individual’s health [63]. In the Republic of Cyprus, asylum seekers are excluded from the national guaranteed minimum income; they receive instead allowances in kind and/or vouchers and a small amount of cash that can be used for utilities and other expenses [62]. Yet, the voucher system is considered as problematic, causing more problems than solutions [62]. Asylum seekers’ experience of financial hardship was also reported in the United Kingdom where the majority of asylum seekers are needy upon arrival [61]. A recent qualitative study on asylum seekers’ experiences with living conditions in Eastern Germany also highlights women’s perceived lack of resources and multitude of socio-environmental challenges during the asylum seeking process [59]. Disposing of limited financial means is burdening for asylum seekers and results in limited consumption potential, a social determinant of health at the meso level, with perceived negative consequences during pregnancy and early motherhood.

In addition, housing and neighbourhood quality have been shown to be a crucial determinant of health [31, 63]. Housing was found to be important to both physical and mental health for asylum seekers [31, 35]. Accommodation affects health and wellbeing via physical characteristics e.g. the housing condition and social characteristics such as safety and security [35]. As stated by the WHO’s *Health Principles of Housing* [64], adequate housing should minimize adverse factors and protect vulnerable populations such as pregnant women. The operational standards of reception centres in Europe, published by the European Asylum Support Office, declare to “ensure respect for the privacy of the applicants in collective housing” [23]. Yet, the detailed statement states a maximum of six single applicants in one bedroom [23], raising doubt on the level of privacy for each individual. Even though host countries should strive for creating a safe environment for asylum seekers [65], e.g. in the United Kingdom, asylum seekers face depriving and insecure housing [33]. As a consequence of the policy of sharing accommodation, often with unknown people, privacy is largely lost [16]. Pregnant women experienced living in a shared room with a stranger as challenging, especially during pregnancy [34]. A study of female refugees in Germany showed that women considered state-provided accommodation with overcrowded space and limited privacy as difficult to endure [36]. Asylum seeking women who were exposed to violence should be availed a protected living environment in order to begin to process their trauma [66]. Yet, reception centres with insufficient privacy, lighting and a lack of separate accommodation for women who are single, may even facilitate exposure to adverse events including aggressions [30]. Therefore, it is important to make sure that reception centres offer well-lit accommodation for women who are single, which they can lock [30]. As poor housing has been identified as having a negative impact on the health and well-being of asylum seekers in a variety of ways, improved quality and safety of housing may lead to better health outcomes [35]. Additionally, hygiene plays a decisive role in housing quality as poor hygiene may have negative effects on health, especially if water and sanitation are not easily accessible [32]. Social determinants of health, decent standards in housing, including hygiene and safe food preparation for occupants’ nutritional status and immunity were already highlighted by the WHO’s *Health Principles of Housing* [64] three decades ago. Housing and neighbourhood quality, as social determinants of health at the micro level, play a substantial role in physical and mental health of asylum seekers and are of even greater importance for those who are pregnant and new mothers.

Another aspect of great importance for women in our study was nutrition. This is backed by latest results from a systematic review and meta-analysis that clearly state the importance of maternal diet quality during pregnancy on outcomes in children [67]. Standards published by the European Asylum Support Office also highlight the relevance of ensuring asylum seekers’ access to adequate food in sufficient quantity in reception centres [23]. Yet, our study participants experienced the prohibition to cook in reception centres and catering services that lacked healthy food and were not sensitive to cultural and religious practices. Similar findings are reported in a study on accommodation centres in the Czech Republic, where asylum seekers considered reception centers’ catering services as particularly oppressive and considered it demeaning, to be unable to control their lives in such an intimate matter as dietary intake [60]. Similar to our findings, poor quality, limited variety and tastelessness of meals offered by catering services were criticised [60]. These findings clash with women’s need of a balanced diet with a sufficient amount of vitamins as this is considered a modifiable risk factor to prevent adverse birth outcomes [68]. As a social determinant of health at the micro level, availing a healthy diet for pregnant women and new mothers living at state-provided reception centres is a basic requirement for health of both mother and unborn child.

Furthermore, pregnant women and new mothers perceived a lack of information and offers of healthy physical activities in reception centres which was considered as troublesome for their health and wellbeing. This stands in contrast to the right to prevention which includes prevention and education programs for behaviour-related health problems and the promotion of social determinants of good health [69]. Implementing policies in all European Union member states that avail separate accommodation, supplementary intermediate meals, healthy sports or activities outside the centre or cooking possibilities for vulnerable persons [19], including pregnant women and single parents, might lead to a higher level of health and wellbeing of these women. Implementing policies and educational programs on health promotion, including healthy sports activities during pregnancy, may enhance women’s health via adjusting the social determinants of health on a macro level.

As health is a basic human right and its realisation may be pursued via the formulation of appropriate health policies, a main objective in promoting women’s right to health should be to reduce women’s health risks, in particular to decrease maternal mortality and to protect women from domestic violence [69]. Yet, there is still a considerable heterogeneity in both EU member states and the German federal states in taking the specific situation and needs of vulnerable persons, including pregnant women and new mothers into account. As declared in a directive by the EU parliament, public policies of member states shall consider the particular situation of vulnerable persons [70]. This includes to consistently provide separate accommodation, offer the possibility to cook or to receive additional intermediate meals and to avail sports possibilities or activities outside the centre, special terms that some EU Member States have already implemented [19]. Therefore, implications for policy makers are that immigration policies should consider material circumstances and behavioural factors as social determinants of health to address the health needs of asylum seekers, including pregnant women and new mothers. Implications for the research community are that further data is needed to provide both quantitative and qualitative evidence in order to meet the health needs of these women including access to, provision of and equity of care. In addition, future studies that include a perspective on human rights and gender issues could further improve the health and wellbeing of this vulnerable group. Such future research would contribute to a sound evidence base for decision makers and help raise awareness of the special risks and health needs of these women.

One strength of this exploratory case study is its in-depth focus on the effects of social determinants of health on pregnant women and new mothers seeking asylum in Germany to illuminate the gained experiences and perceived needs with the accompanying consequences for women’s health and wellbeing whilst being accommodated at a state-provided reception center or an accommodation center. Acknowledging that this is a qualitative study on asylum seeking women in one federal state in Southern Germany and results are therefore not generalisable to a broader population, there may be common aspects for asylum seeking women during pregnancy and early motherhood, which are to health care providers and researchers in other areas. Additionally, the consolidated criteria for reporting qualitative studies and the standards for reporting qualitative research were used whilst preparing results for publication to maximize trustworthiness and to ensure the quality of our findings. Therefore, the findings of this study must be considered under the specific quality criteria applying to qualitative research that were applied in this study. A further strength of our study can be found in the conceptual framework of the WHO [3] which supported our data analysis, as this can facilitate international comparisons and research endeavours in the related field for other researchers.

Nevertheless, some limitations must be considered when interpreting these results. A limitation of this study could have been the 20 Euro compensation for study participation during each interview. As financial hardship was reported by many participants, the compensation might have been an incentive to participate in the study. In addition, professional interpreting services have enabled us to overcome language barriers, but there is a risk of losing information caused by interpretations and it is not known how these interpreting services influenced the statements of the participants that formed the basis of the analysis. Based on the fact that there were nine study participants, the results must be interpreted with caution. Since the results are from a specific context, it should be taken into account that the results of a similar study carried out elsewhere may be very different. However, this small sample size allowed us to collect detailed data. Despite these limitations, the unique findings of our study contribute to the evidence base available for generating health-related policies and inform healthcare providers about this vulnerable group. Further studies examining the health-related consequences of state-provided accommodation and influencing social health determinants are needed, using both qualitative and quantitative approaches. These aspects must be considered when interpreting these results of this study.

## Conclusions

Pregnant women and new mothers seeking asylum in Germany experienced significant health challenges due to major constraints in material circumstances as social determinants of health whilst living in state-provided accommodation. This included the experiences of a poor housing and neighbourhood quality with a lack of privacy, security and hygiene at state-provided reception centres, causing fear to impair the course of a healthy pregnancy. Restrictive policies at state-provided reception centres, such as the prohibition to cook, were considered as negatively affecting women’s health, especially during pregnancy. Direct provisions’ catering services were perceived as insufficient and ignoring religious practices. A minimal living allowance severely limited women’s consumption potential. Women considered these major restrictions in material circumstances as detrimental to health during pregnancy and early motherhood.

## Additional files

Additional file 1: Interview Guide for first interview during pregnancy. (DOCX 19 kb)

Additional file 2: Interview guide for the second interview during pregnancy. (DOCX 19 kb)

Additional file 3: Interview guide for third interview after delivery. (DOCX 19 kb)

## Abbreviations

AC: Accommodation centre
COREQ: Consolidated criteria for reporting qualitative studies
CSDH: Commission on Social Determinants of Health
EU: European Union
ID: Identification
M: Married
RC: Reception centre
S: Single
SDH: Social determinants of health
SP: Study participant
SRQR: Standards for reporting qualitative research
T 1: Interview 1 (prenatal)
T 2: Interview 2 (prenatal)
T 3: Interview 3 (postnatal)
WHO: World Health Organization
