# Supplementary material for: “If you can, change this system” -Pregnant asylum seekers‘ perceptions on social determinants and material circumstances affecting their health whilst living in state-provided accommodation in Germany - a prospective, qualitative case study
Source: BMC Public Health. 2019 Mar 12;19:287. doi: 10.1186/s12889-019-6481-2 (PMC6417255; doi:10.1186/s12889-019-6481-2)
Supplement: Supplementary file 1 — Interview Guide for first interview during pregnancy. (DOCX 19 kb) [file 12889_2019_6481_MOESM1_ESM.docx]

**Additional file 1)** **Interview Guide for first interview during pregnancy**

| Topics | Subtopics |
| --- | --- |
| 1. General wellbeing | - How do you feel today? |
| 1. Living environment | - What do you think about living in this place (reception centre)? - When did you arrive? - What happened since arrival? - Accommodation? Transfers? - Describe your living situation/daily routine - What do you do?   - How do you feel about your accommodation? What would you like to change? What are your thoughts about a transfer? What do you think would change after a transfer?   - How many other people in room? Privacy? Sleep?   - How is the relationship with the room neighbours?   - Bathroom, hygienic? Share with how many?   - Food – quality? Amount? Religious restrictions such as halal?   - With whom do you eat?   - When/ how often do you leave your accommodation facility? Public transportation   - Money? What do you pay? - Autonomy / self-responsibility?   - Actively search for answers vs. waiting for information - Contact with others? Inside and outside accommodation? Other asylum-seekers and people working at the facility - Does the accommodation influence your wellbeing and health? How? What should be different? |
| 1. Pregnancy | - How does it feel to become a mother? - First pregnancy? - Since when do you know that you are pregnant?   - Where did you find out?   - Feelings when you found out? - What changed because of the pregnancy?   - Any actions taken? - Feelings now/ expectations   - Can you feel the baby already?   - Estimated date of delivery? - How is your relationship with the father of your baby? Do you live together? |
| 1. Medical care in pregnancy | - Which healthcare providers did you see? Why? - Experiences with midwife or gynecologist?   - Differences     - Describe your visit     - Gender of provider/interpreter   - Did you see a women’s doctor? (if no, continue with midwife only; if yes, first doctor than midwife)   - How many midwives/doctors?   - How did you know about it?     - Who initiated your visit? Who made your appointment?     - Did you want to see a doctor/midwife? Why?     - Transportation/ challenge to find the place?     - Who was with you?   - How did you feel when seeing a doctor?     - Did you feel well treated? /cared for/ respected?   - What did she examine?   - How was the communication?     - Language? Who helped out?     - Did you ask any questions? Articulation of wishes/preferences/fears   - Can you share your experience with the waiting time to meet doctor/midwife?   - Which questions did you ask during the consultation? Which answers did you get?   - May I see your maternity record (document where midwives and gynecologists note test results pre- and postnatal on mother’s and infant’s health status) - Expectations/needs   - Do you get what you need to be healthy?   - What did you expect from doctor/midwife?     - Anything you did not get? – Example?   - Would you like to go there again? Why? Why not? How often? Do you have another appointment? Midwife/women’s doctor? |
| 1. Personal characteristics | - Age - First pregnancy? - Single/Relationship/Married? - Where did you live before arriving here? - What do you consider your home country? When did you leave it? - Religious? - With whom did you come here? By yourself? Family members? - For how many years did you go to school? - What is your profession? For how long did you work? - Which languages do you speak? |
| 1. Additions | - Would you like to ask me anything? - Do you have any general questions? Or remarks? - Would you like to share something that I haven’t asked you? |
