# Supplementary material for: “If you can, change this system” -Pregnant asylum seekers‘ perceptions on social determinants and material circumstances affecting their health whilst living in state-provided accommodation in Germany - a prospective, qualitative case study
Source: BMC Public Health. 2019 Mar 12;19:287. doi: 10.1186/s12889-019-6481-2 (PMC6417255; doi:10.1186/s12889-019-6481-2)
Supplement: Supplementary file 3 — Interview guide for third interview after delivery. (DOCX 19 kb) [file 12889_2019_6481_MOESM3_ESM.docx]

**Additional file 3) Interview guide for third interview after delivery**

| 1. General wellbeing | - How do you feel today? Why? - What happened since last interview? |
| --- | --- |
| 2. Living environment | - Any changes in your accommodation? In living environment?   - How do you feel about your accommodation? What would you like to change? What are your thoughts about a transfer? What do you think would change after a transfer?   - Privacy? Sleep?   - How is the relationship with the room neighbours?   - Food?   - How is your financial situation?   - Contact with others? Inside and outside accommodation? - Does the accommodation influence your wellbeing and health? How? What should be different? |
| 3. Delivery | - Congratulations to the birth of your child. How does it feel to be a mother? - When did you give birth? - Any difficulties? - Expectations/fears/worries before delivery - Please tell me what happened when the labor pain/contractions started (how did you get to the hospital? Who called taxi/ambulance? When exactly did you go there? Labor?) - How was the situation for the father of the baby? - Did you choose the hospital? - How do you feel now? - How was the care in the hospital? Who was with you? For how long did you stay? - Did you feel you were treated differently than other patients? How? - How did you communicate with others? (Language barrier?) |
| 4. Medical care in pregnancy/post-partum | - Experiences with midwife or gynecologist   - Did you see a women’s doctor before/after delivery? (if no, continue with midwife only; if yes, first doctor than midwife)   - How many midwives/doctors? (same person twice?)   - How did you know about it?     - Who initiated your visit? Who made your appointment?     - Did you want to see a doctor? Why?     - Transportation/ challenge to find the place?     - Who was with you?     - Helpful?   - How was the communication?     - Language? Who helped out?     - Did you ask any questions? Articulation of wishes/preferences/fears   - Are you planning on seeing a doctor/midwife again? Why? Any appointments?   - Maternal health record /children’s examination booklet - Was there anything that you needed that you did not receive? Can you explain? |
| 1. Additions | - Would you like to ask me anything? Do you have any general questions? Or remarks? Would you like to share something that I haven’t asked you? |
